# Phase II study of apatinib plus exemestane in estrogen receptor-positive, human epidermal growth factor receptor 2-negative metastatic breast cancer

**DOI:** 10.1080/15384047.2023.2265055

**Published:** 2023-10-13

**Authors:** Aihua Tan, Li Nong, Hongxue Wang, Yuxian Jia, Wuning Zhong, Fanghui Qin, Han Wang, Jing Tang, Yan Liu, Yongkui Lu

**Affiliations:** Department of Breast, Bone & Soft Tissue Oncology, Guangxi Medical University Cancer Hospital, Nanning, Guangxi, China

**Keywords:** Apatinib, exemestane, metastatic breast cancer (MBC), endocrine resistance

## Abstract

**Purpose:**

Apatinib is a tyrosine kinase inhibitor targeting vascular endothelial growth factor receptor (VEGFR)-2. This study was conducted to assess the efficacy and safety of apatinib combined with exemestane in patients with estrogen receptor-positive (ER+)/human epidermal growth factor receptor 2-negative (HER2-) metastatic breast cancer (MBC).

**Methods:**

This single-center, single-arm phase II study enrolled patients with ER+/HER2- MBC progressed on previous letrozole or anastrozole. Stratified analysis was performed according to the number of chemotherapy regimens for metastatic disease. The primary endpoint was progression free survival (PFS). Secondary endpoints included objective response rate (ORR), disease control rate (DCR), clinical benefit rate (CBR), overall survival (OS) and toxicity. Patients received apatinib at a starting dose of 500 mg/d and exemestane 25 mg/d on days 1–28 of each 4-week cycle.

**Results:**

Thirty patients were enrolled with median four prior anticancer therapies. Eighty percent of patients received chemotherapy for metastatic disease. The median PFS (mPFS) and OS were 5.6 (95%CI: 4.3–6.9) months and 15.7 (95% CI: 9.7–21.7) months, respectively. The ORR, DCR, and CBR were 21.4%, 71.4%, and 46.4%, respectively. Patients with 0–1 line chemotherapy for MBC showed a slightly longer mPFS compared to those with ≥2 lines chemotherapy (mPFS: 6.4 months vs 4.8 months, *P* = .090). Most of the AEs were grade 1/2. One patient (3.3%) who suffered bone marrow metastases experienced grade 4 thrombocytopenia, and 14 experienced grade 3 AEs. Fifty percent of patients were given reduced dose for apatinib.

**Conclusions:**

Apatinib plus exemestane exhibited objective efficacy in patients with ER+/HER2- MBC who have failed multiple lines of treatment. The AEs of apatinib required close monitoring and most of patients were well tolerated.

## Introduction

Nearly 70% of breast cancer is estrogen receptor-positive (ER+), human epidermal growth factor receptor 2-negative (HER2-).^1^ Although effective therapy for early breast cancer has reduced the 5-year recurrence rates and mortality rates, about 20% patients still experience disease relapse or metastasis.^2^ For patients with ER+/HER2- metastatic breast cancer (MBC), endocrine therapy and chemotherapy are both important treatment options. However, unfortunately, nearly all patients with MBC will eventually develop drug resistance, and new therapeutic options for such patients are urgently needed.

Much evidence supports a close interaction of the ER signaling with angiogenesis and its vascular endothelial growth factor receptor (VEGFR) signaling pathway.^3–5^ Angiogenesis is influenced by estrogen under both physiological and pathological conditions, and vascular endothelial growth factor (VEGF) and its receptors play a crucial role in the growth of blood vessels.^6–8^ Takei H et al.^9^ reported that estrogen stimulated both the growth rates and VEGF production of ER+ breast cancer cells (MCF-7). Nakamura J et al.^10^ reported that estrogen acting as a mammary tumor promotor regulated vascular endothelial growth/permeability factor (VEG/PF) expression in 7,12-dimethylbenz(a)anthracene-induced rat mammary tumors, leading to increased tumor angiogenesis and/or permeability of the microvessels to allow tumor cell migration. The most revealing evidence of the relationship between angiogenesis and endocrine comes from allograft data.^11^ After castration, the tumor volumes are significantly reduced and the tumoral vascularity lowered in androgen-dependent male mouse models. When endocrine resistance occurred, a large number of new blood vessels are regenerated and the tumors enlarge. At the molecular level, the expression of VEGF mRNA decreases after castration, accompanied by tumor shrinkage and vascular regression. Moreover, it is interesting that the apoptosis of endothelial cells precede tumor cells after castration, which supports that endocrine therapy has a direct effect on tumor vascularization. In patients with breast cancer, retrospective studies demonstrate that increased tumor VEGF levels are associated with decreased responsiveness to antiestrogen therapy and worse outcome.^12,13^ Together, these results suggest that VEGF and the VEGFR pathway may be an important target for treatment of ER+ MBC and antiangiogenic agents could be more effective in a low-estrogen environment.

Apatinib, a small-molecule VEGFR-tyrosine kinase inhibitor, selectively acts on the intracellular ATP binding site of VEGFR-2, which plays a central role in the regulation of angiogenesis.^14^ It blocks VEGF-VEGFR downstream signal transduction, leading to tumor angiogenesis inhibition. Besides, higher concentrations of apatinib also inhibit PDGFR β, C-KIT and C-Src kinases. Preclinical studies have demonstrated that apatinib has a good efficacy and high tumor inhibition rate in the treatment of human xenograft tumors in nude mice.^15^ Subsequent phase II clinical trials^16,17^ reported that apatinib monotherapy achieved clinical effects in metastatic triple-negative BC (TNBC) or non-TNBC who have failed multiple lines of therapy, and the safety was controllable, which supported further clinical evaluation of apatinib in the treatment of MBC.

At present,apatinib has been approved as third-line treatment for patients with advanced gastric adenocarcinoma in China, October 2014.^18^ Clinical trials of apatinib for other cancer types are under the way, and there is no trial to evaluate the combination of apatinib with endocrine therapy. This study was conducted to evaluate the efficacy and safety of apatinib combined with exemestane in the treatment of ER+/HER2- MBC.

## Methods

### Patients

The included patients aged 18–70 years and were pathologically diagnosed as MBC, with ER+ (+, >10%) and HER2–. HER2- was defined as no staining or scores of 1+ by immunohistochemistry (IHC), and cases with 2+ by IHC were confirmed absence of HER2 gene amplification by fluorescence in situ hybridization (FISH). Patients were either postmenopausal women or receiving ovarian suppressive therapy. All patients had experienced treatment failure with previous anastrozole or letrozole, defined as disease recurrence or metastasis during or within one year after the end of adjuvant endocrine therapy, or progression during endocrine therapy in MBC. Chemotherapy or other endocrine therapy (tamoxifen, fulvestrant, toremifene, progesterone) for advanced disease were also allowed before inclusion. Patients who had previously taken exemestane were excluded unless they had experienced disease recurrence or metastasis one year after the end of adjuvant therapy with exemstane. The included patients had to have at least one measurable lesion, a life expectancy of no less than 3 months, and an Eastern Cooperative Oncology Group (ECOG) performance status of 2 or less. They also had to have adequate hematologic, hepatic, and renal function, as indicated by hemoglobin ≥8 g/dL, absolute neutrophil count (ANC) ≥1.5 × 109/L, platelet count ≥75 × 109/L, total serum bilirubin ≤1.5 × upper limit of normal (ULN), AST/ALT ≤2.5 × ULN (≤5× ULN in case of liver metastases), and serum creatinine ≤1.0 × ULN (calculated creatinine clearance ≥50 mL/min). Patients with symptomatic brain metastases, uncontrolled hypertension, serious cardiovascular disease or previous treatment with angiogenesis inhibitors were excluded. At last, a total of 30 patients were enrolled in this study. All of the enrolled patients signed the informed consent and could be cooperative with medical workers. The study was approved by the Medical Ethics Committee of Guangxi Medical University Cancer Hospital (KS2017(05)) and was conducted in accordance with the Declaration of Helsinki (as revised in 2013). The study protocol has been registered in the Chinese Clinical Trial Registry (Identifier: ChiCTR-OIC-17010440)

### Intervention measures

Eligible patients received apatinib at a starting dose of 500 mg/d and exemestane 25 mg/d on days 1–28 of each 4-week cycle. Apatinib could also be started with 250 mg/d, and then gradually increased to 500 mg/d in one week, as to improve tolerance for the old and infirm patients. A dose reduction of apatinib will be allowed to 250 mg/d. If grade 3/4 hematological toxicity or nonhematologic adverse events (AEs) such as hand and foot syndrome, hypertension, proteinuria, etc occur for the first time, it is recommended to stop the drug temporarily until the AEs relieve or disappear. If grade 3/4 of the above AE occurs again after resuming the drug, the drug can be continued after lowering the dose. If the AEs still persist, the drug will be stopped. The above regimen was performed until progressive disease, unacceptable toxicity, or consent withdrawal, whichever came first.

### Baseline and follow-up assessment

The pretreatment evaluation included detailed medical history, physical examination, routine laboratory tests, and computed tomography (CT) of measurable lesions at baseline. Laboratory tests included blood routine, liver function, renal function, electrolytes, and urinalysis. CT was conducted for efficacy evaluation every two cycles for the first 4–6 cycles, and then every 2–3 months until disease progression. Adverse events (AEs) were recorded at each cycle until 30 days after the last dose therapy. Subjects were observed until death, loss to follow-up, withdrawal from the trial, or the end of this study.

### Clinical efficacy

Efficacy was evaluated according to the Response Evaluation Criteria in Solid Tumors 1.1 (RECIST 1.1),^19^ and was divided into complete response (CR), partial response (PR), stable disease (SD) and progressive disease (PD). Progression-free survival (PFS) was defined as the time from patient enrollment to the first recorded disease progression or death (whichever occurs first). Overall survival (OS) was defined as the time from patient enrollment to death (from any cause of death). The objective response rate (ORR) was calculated as CR+PR, the disease control rate (DCR) was calculated as CR+PR+SD, and the clinical benefit rate (CBR) was calculated as (CR+PR+SD)>6 m. PFS was the primary endpoint and ORR, DCR and CBR were serving as secondary endpoint. Treatment-related AEs were assessed and graded according to the National Cancer Institute Common Terminology Criteria for AEs (Version 4.0).^20^

### Statistical analysis

In sample size estimate, 24 months of accrual period and 6 months of follow-up period were assumed. The study was designed with two-sided, α = 0.05, 85% power to detect a null median PFS of 2.8 months and experimental median PFS of 5.6 months (*n* = 25). Assuming a 20% dropout rate, final accrual number was 30.

All statistical analyses were conducted by using SPSS version 18 (SPSS Inc., Chicago, IL). The categorical data was described as frequency or percentage (%), and the chi-square test or Fisher exact probability method was used for comparison between groups. Kaplan–Meier method and log-rank test were used for survival analysis. The stratified analysis was performed according to the number of chemotherapy lines for advanced disease. *P* < .05 was considered significant.

## Results

### General information

**Figure 1.**
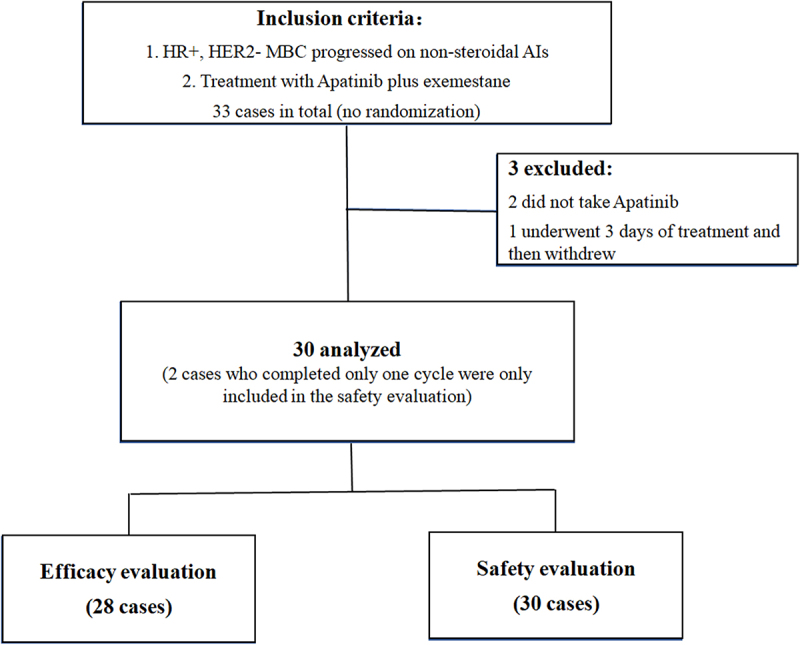
Flowchart of the participants through the trial (CONSORT diagram).

A total of 33 patients with ER+/HER2- MBC progressed on previous treatment of anastrozole or letrozole in the Guangxi Medical University Cancer Hospital were recruited between April 2017 and November 2019. Two cases who did not take apatinib and one who only underwent 3 days of treatment were excluded. Finally, 30 cases were included in the analysis (Figure 1) and their characteristics are listed in Table 1. Thirty patients were all females and their age ranged 33–70 years (median age, 47.0 years). Among them, 22 (73.3%) patients had ≥3 metastatic organ sites, and visceral involvement was noted in 26 ones (86.7%). Twenty-four (80%) patients received chemotherapy in metastatic setting and the number of treatment lines for chemotherapy ranged 0–6 (median: 2). Twelve (40.0%) patients have received 0–1 chemotherapy regimens and 18 (60.0%) have received two or more chemotherapy regimens for metastatic disease. Besides, 11 (36.7%) patients had previously received Fulvestrant endocrine therapy, and 2 (6.7%) patients received CDK4/6 inhibitor or Everolimus targeted therapy, respectively (Table 1).

**Table 1.** Patient characteristics (*N* = 30).

Characteristics	Number	%
Age, years		
Median (Range)	47.0(33.0–70.0)	
ECOG status		
0–1	19	63.3
2	11	36.7
Hormone Receptor Status		
ER+/PR+	26	86.7
ER+/PR-	4	13.3
Sites of Metastasis		
Visceral	26	86.7
Bone only	1	3.3
Soft tissue only	3	10.0
No. of metastatic sites		
1	2	6.7
2	6	20.0
≥3	22	73.3
Previous treatment with letrozole or anastrozole	30	100.0
Previous treatment with antiestrogen		
Any antiestrogen	22	73.3
Tamoxifen	15	50.0
Fulvestrant	11	36.7
No. of previous endocrine therapy for metastatic disease		
0–1	15	50.0
2	9	30.0
3	6	20.0
Previous chemotherapy		
Neoadjuvant or adjuvant therapy only	6	20.0
Treatment of metastatic disease	24	80.0
Chemotherapy lines for metastatic disease, Median(Range)	2（0–6）	
No. of chemotherapy lines for metastatic disease		
0–1	12	40.0
≥2	18	60.0
Prior chemotherapy drugs in metastatic setting		
Anthracycline	9	30.0
Taxane	14	46.7
Gemcitabine/Capecitabine/Vinorelbin	19	63.3
Platinum	18	60.0
Etoposide/Cyclophosphamide	9	30.0
Pemetrexed	9	30.0
Previous targeted therapy		
CDK4/6 inhibitor	1	3.3
Everolimus	1	3.3

### Clinical efficacy

**Figure 2.**
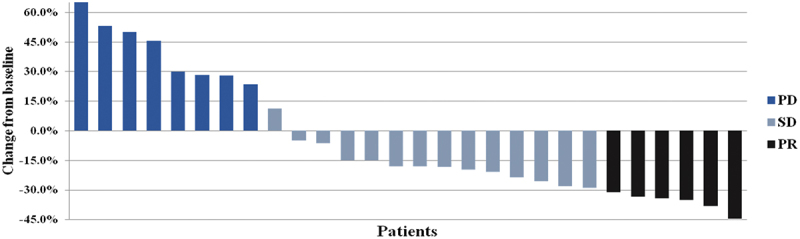
Best overall response. PR, partial response; SD, stable disease; PD, progression disease.

**Figure 3.**
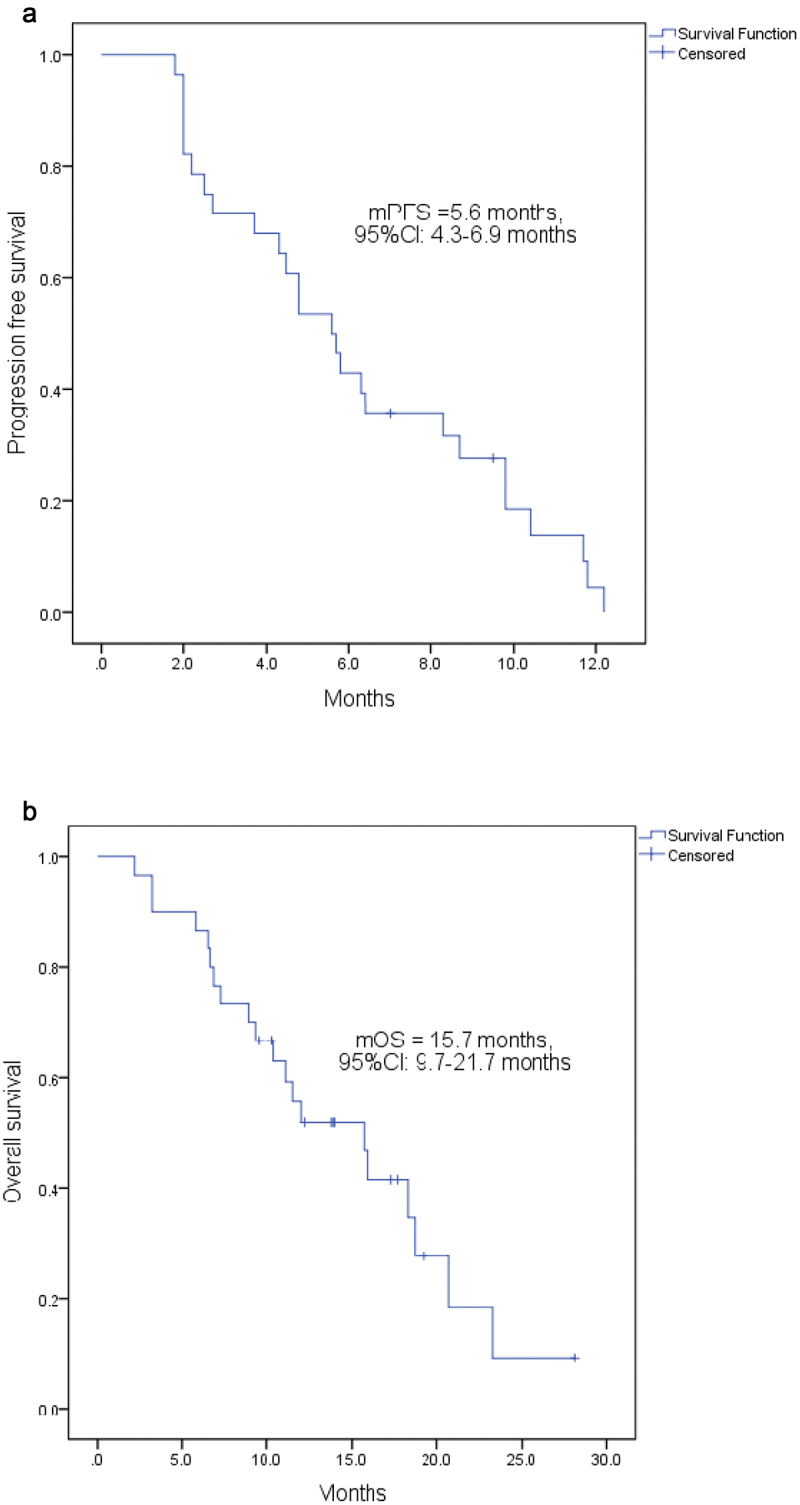
Kaplan-–Meier estimates. (a) the overall PFS; (b) the OS. PFS, progression-free survival; OS, overall survival; mPFS, median PFS.

Treatment efficacy is summarized (Table 2, Figure 2). Of the 30 enrolled subjects, 28 patients who completed at least one full treatment cycle were included in the efficacy evaluation, and the remaining 2 subjects who completed less than one cycle were only included in the safety evaluation (Table 3). Among the 28 patients, no one achieved CR. However, 6 patients (21.4%) achieved PR and 14 (50.0%) achieved SD. Thus, the ORR and DCR were 21.4% and 71.4%, respectively. Besides, 13 patients had stable or remission of disease for more than 6 months, and the CBR was 46.4%. The last follow-up date was August 30, 2020. The median PFS was 5.6 (95% CI: 4.3–6.9) months (Figure 3a) and the median OS was 15.7 (95% CI: 9.7–21.7) months (Figure 3b).

**Table 2.** Summary of treatment efficacy.

Efficacy	Total (*n* = 28)	^a^Patients with 0–1 line chemotherapy for MBC (*n* = 12)	^b^Patients with ≥ 2 lines chemotherapy for MBC (*n* = 16)	^#^*P* value
CR	0 (0.0%)	0 (.0%)	0 (.0%)	–
PR	6 (21.4%)	4 (33.3)	2 (12.5)	–
SD	14 (50.0%)	6 (5.0)	8 (5.0)	–
PD	8 (28.6%)	2 (16.7)	6 (37.5)	–
ORR (CR+PR)	6 (21.4%)	4 (33.3)	2 (12.5)	.354
DCR (CR+PR+SD)	20 (71.4%)	10 (83.3)	10 (62.5)	.401
CBR (CR/PR/SD >6 m)	13 (46.4%)	7 (58.3)	5 (31.3)	.250
PFS, median (95%CI), m	5.6 (4.3–6.9)	6.4 (1.8–11.0)	4.8 (3.8–5.8)	.090
OS, median (95%CI), m	15.7 (9.7–21.7)	15.9 (6.7–25.1)	12.0 (6.4–17.6)	.343

MBC, metastatic breast cancer; CR, complete response; PR, partial response; SD, stable disease; PD, progressive disease; ORR, objective response rate; DCR, disease control rate; CBR, clinical benefit rate; PFS, progression-free survival; OS, overall survival.

^#^statistical analysis between group a and b.

**Table 3.** Adverse events (*N* = 30).

AEs	All Grades n (%)	Grade 1 n (%)	Grade 2 n (%)	Grade 3 n (%)	Grade 4 n (%)
**Hematologic**					
Thrombocytopenia	2(6.7)	1(3.3)	0	0	1(3.3)
Anemia	2(6.7)	2(6.7)	0	0	0
Leukopenia	3(10.0)	2(6.7)	1(3.3)	0	0
**Nonhematologic**					
Hypertension	22(73.3)	9(30.0)	9(30.0)	4(13.3)	0
Proteinuria	22(73.3)	15(50.0)	4(13.3)	3(10.0)	–
Hand-foot syndrome	27(90.0)	11(36.7)	13(43.3)	3(10.0)	–
Fatigue	25(83.3)	22(73.3)	2(6.7)	1(3.3)	–
Pain	17(56.7)	11(36.7)	5(16.7)	1(3.3)	
Apositia	10(33.3)	9(30.0)	1(3.3)	0(0.0)	0(0.0)
Nausea/vomiting	7(23.3)	6(20.0)	1(3.3)	0(0.0)	0(0.0)
Rash	3(10.0)	1(3.3)	1(3.3)	1(3.3)	0(0.0)
Mucositis	15(50.0)	9(30.0)	5(16.7)	1(3.3)	0(0.0)
Dizzy	8(26.7)	8(26.7)	0(0.0)	–	–
Elevated transaminase	6(20.0)	5(16.7)	1(3.3)	0(0.0)	0(0.0)
Hemorrhage	3(10.0)	3(10.0)	0	0(0.0)	0(0.0)
Fever	1(3.3)	1(3.3)	0	0(0.0)	0(0.0)
Abdominal distension	4(13.3)	3(10.0)	1(3.3)	0(0.0)	0(0.0)
Diarrhea	3(10.0)	3(10.0)	0(0.0)	0(0.0)	0(0.0)

AEs, adverse events.

Hemorrhage: Oral bleeding (1), Vaginal bleeding (1), Bleeding from a breast mass (1).

**Figure 4.**
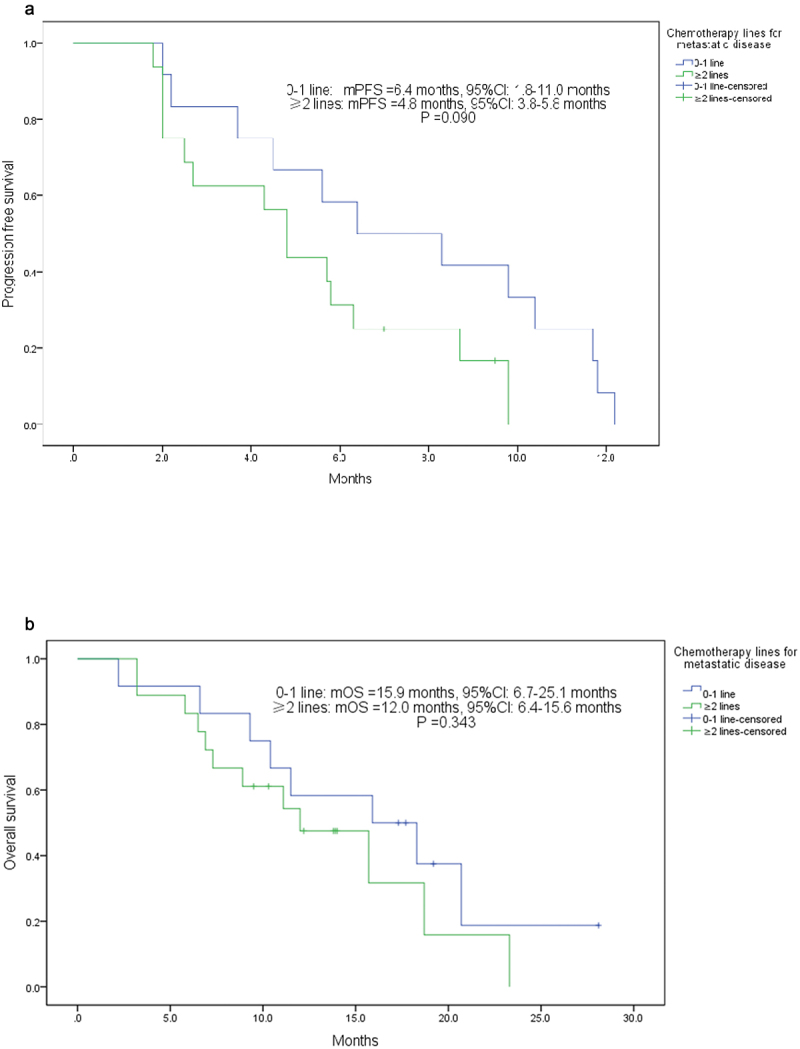
The stratified analysis. (a) the mPFS of patients with 0–1 line or ≥ 2 lines chemotherapy for MBC; (b) the OS of patients with 0–1 line or ≥ 2 lines chemotherapy for MBC. PFS, progression-free survival; OS, overall survival; mPFS, median PFS; MBC, metastatic breast cancer.

In order to evaluate the effect of chemotherapy on the treatment of apatinib plus exemestane, stratified analysis was performed according to the number of chemotherapy lines (0–1 lines of chemotherapy vs ≥2 lines of chemotherapy). Patients with 0–1 line chemotherapy for MBC had an ORR of 33.3%, DCR of 83.3%, CBR of 58.3%, respectively, higher than those reported in patients with ≥2 lines chemotherapy for MBC who had an ORR of 12.5%, DCR of 62.5%, CBR of 31.3%, respectively. However, no significant differences were observed because of the small sample size (all *P* > .05) (Table 2). For survival analysis, patients with 0–1 line chemotherapy for MBC had a longer PFS of 6.4 (95% CI: 1.8–11.0) months than that in patients with ≥2 lines chemotherapy for MBC who had a PFS of 4.8 (95% CI: 3.8–5.8) months, and the difference nearly reached statistical significance (*P* = .09) (Figure 4a). Patients with 0–1 line chemotherapy or ≥2 lines chemotherapy for MBC had a OS of 15.9 (95% CI: 6.7–25.1) months and 12.0 (95% CI: 6.4–15.6) months, respectively. No statistically significant difference was observed (*P* = .343) (Figure 4b).

### Toxic effects

The drug-related adverse events (AEs) are presented in Table 3. The most frequent AEs included hand-foot-skin reaction (*n* = 27, 90.0%), fatigue (*n* = 25, 83.3%), hypertension (*n* = 22, 73.3%), proteinuria (*n* = 22, 73.3%), pain (*n* = 17, 56.7%), mucositis (*n* = 15, 50.0%), apositia (*n* = 10, 33.3%), dizzy (*n* = 8, 26.7%), and nausea/vomiting (*n* = 7, 23.3%). Most of the AEs were grade 1/2. One patient (3.3%) who suffered bone marrow metastases experienced grade 4 thrombocytopenia, and 14 experienced grade 3 AEs, mainly hypertension (*n* = 4, 13.3%), hand-foot-skin reaction (*n* = 3, 10.0%), proteinuria (*n* = 3, 10.0%), fatigue (*n* = 1, 3.3%), pain (*n* = 1, 3.3%), rash (*n* = 1, 3.3%), and mucositis (*n* = 1, 3.3%). Two patients (6.7%) withdrew the trial because of the AEs. One patient withdrew the trial because of the grade 4 thrombocytopenia, and the second patient withdrew the trial because of the persistent rash and fatigue, even when apatinib dose was reduced to 250 mg Qd. Due to the intolerant AEs, mainly hand-foot-skin reaction, proteinuria, fatigue, pain, and rash, 15 (50%) patients were treated with dose reduction for apatinib.

## Discussion

Endocrine therapy is a standard of care for ER+/HER2- MBC, and chemotherapy is the second approach. Unfortunately, resistance eventually develops in MBC and becomes a major clinical challenge. There is a need to find new therapeutic strategies to improve treatment outcomes. Angiogenesis promotes tumor invasion and metastasis,^21^ and the induction of VEGF is implicated as a mechanism for the emergence of endocrine therapy resistance. Apatinib is a small-molecule tyrosine kinase inhibitor that selectively acts on VEGFR2, and its effect on the reversal of endocrine resistance remains unclear. Our study was the first to evaluate apatinib in combination with endocrine agents in ER+/HER2- MBC. The patients included in our study were heavily pretreated with a median of four prior lines of therapy in the MBC setting, 50% having received ≥2 prior endocrine therapy and 60% having received ≥2 prior chemotherapy. In this poor prognostic setting, apatinib plus exemestane has demonstrated an ORR of 21.4%, CBR of 46.4%, and median PFS of 5.6 months. We further evaluated the effect of prior chemotherapy on the treatment of apatinib plus exemestane, and found that patients with 0–1 line chemotherapy for MBC had an ORR of 33.3%, DCR of 83.3%, and PFS of 6.4 months, respectively. The efficacy of apatinib plus exemestane was better than that of exemestane alone reported by Baselga J et al.^22^ They included patients with ER+/HER2- MBC whose disease was also refractory to previous letrozole or anastrozole, and a single prior chemotherapy regimen for advanced disease was allowed. They reported that patients with exemestane plus placebo had an ORR of 0.4% and PFS of 2.8 months. For patients with ≥2 lines chemotherapy for MBC, apatinib plus exemestane demostrated an ORR of 12.5%, DCR of 62.5%, CBR of 31.3%, median PFS of 4.8 months, and median OS of 12.0 months. These results were similar to the efficacy data of CDK4/6 inhibitors in highly pretreated patients with ER+/HER2- MBC. Ban M et al.^23^ did a retrospecive analysis of 24 heavily pretreated patients with ER+/HER2- MBC and found pateints treated with palbociclib/aromatase inhibitors had a DCR of 58.3%, median PFS of 4.8 months, and median OS of 11 months. Hoste G et al.^24^ included ER+/HER2- MBC progressed on at least four lines of systemic treatment and found that palbociclib in combination with endocrine therapy had a median PFS of 3.17 months and CBR of 41.5%.

The adverse events observed in this study were mainly due to apatinib. The safety profile of apatinib in our study was consistent with that reported in the other breast cancer study,^16,17^ and no new AEs occurred. However, the incidence of common AEs (e.g., hand-foot-skin reaction 90.0%, fatigue 83.3%, hypertension 73.3%, proteinuria 73.3%, pain 56.7%, mucositis 50.0%) was significantly higher than those (e.g., hand-foot-skin reaction 52.6%, fatigue 15.8%, hypertension 42.1%, proteinuria 52.6%, pain 31.6%, mucositis 15.8%) previously reported.^16^ This might be related to the population included in our study. More than half of the subjects in our study had received four or more lines treatment previously, and three patients (10%) had received eight of previous lines of therapy for metastatic disease. While, in the study reported by Hu XC,^17^ the number of previous lines of therapy for metastatic disease ranged 1 to 4, and more than 90% of the subjects had no more than three previous treatment regimens. As we know, the patient’s tolerance to treatment decreases with the duration of treatment. The most frequently observed AEs of apatinib of all grade in this study were non-hematological toxicity, which mainly influenced the treatment compliance with apatinib and reduced patients’ quality of surviving. So, 15 (50%) patients were given dose reduction for apatinib. Hemotologic toxicities including thrombocytopenia, anemia, and leukopeina were basically mild to moderate. Only one patient with bone marrow metastases experienced severe thrombocytopenia and withdrew from treatment. As a result, careful monitoring of toxicity and prompt dose interruption or reduction from 500 mg to 250 mg was essential during the treatment.

### Limitations

Besides, there were several limitations in this study. First, only 30 eligible patients were included in our study, and the subgroup analysis relied on a small number of subjects, which would affect the accuracy of results. Besides, it was of great urgency to explore and unveil alternative treatment option for highly pretreated patients with ER+/HER2- MBC. Hence, only a limited number of clinical cases were involved in this preliminary attempt. Second, this study was a single-arm, single-center, non-blind study with no control group, which could inevitably lead to some biases. Therefore, more clinical cases will be continuously accumulated to conduct larger sample size, multi-center, randomized and controlled clinical trials to provide definitive validation for the use of apatinib plus exemestane as a follow-up strategy against ER+/HER2- MBC.

## Conclusions

This single-arm phase II study demonstrated that apatinib plus exemestane provided a clinical benefit and could be considered as one of the treatment options for women with ER+/HER2- MBC who have failed multiple lines of treatment. However, the AEs of apatinib required close monitoring during treatment, and in general, the vast majority of patients were well tolerated.

## Data sharing statement

The data used and analyzed in the study is available from the corresponding author on reasonable request.

## Reporting checklist

The authors have completed the TREND reporting checklist.
